# The effect of postoperative anticoagulation on acute aortic dissection: a systematic review and meta-analysis

**DOI:** 10.3389/fcvm.2023.1173945

**Published:** 2023-05-10

**Authors:** Xiangfeng Gong, Qianlei Lang, Chaoyi Qin, Wei Meng, Zhenghua Xiao

**Affiliations:** ^1^ Department of Cardiovascular Surgery, West China Hospital, Sichuan University, Chengdu, China; ^2^ Cardiovascular Surgery Research Laboratory, West China Hospital, Sichuan University, Chengdu, China

**Keywords:** acute aortic dissection, postoperative anticoagulation, false lumen, aorta-related death, meta-analysis.

## Abstract

**Background and aim:**

The evolution of the false lumen after the repair of acute aortic dissection has been linked to numerous adverse clinical outcomes, including increased late mortality and a higher risk of reoperation. Despite the widespread use of chronic anticoagulation in patients who have undergone repair for acute aortic dissection, the effects of this therapy on false lumen evolution and its subsequent consequences are yet to be fully understood. This meta-analysis aimed to investigate the impact of postoperative anticoagulation on patients with acute aortic dissection.

**Methods:**

In PubMed, Cochrane Libraries, Embase, and Web of Science, we performed a systematic review of nonrandomized studies, comparing outcomes with postoperative anticoagulation vs. non-anticoagulation on aortic dissection. We investigated the status of false lumen (FL), aorta-related death, aortic reintervention, and perioperative stroke in aortic dissection patients with anticoagulation and non-anticoagulation.

**Results:**

After screening 527 articles, seven non-randomized studies were selected, including a total of 2,122 patients with aortic dissection. Out of these patients, 496 received postoperative anticoagulation while 1,626 served as controls. Meta-analyses of 7 studies revealed significantly higher FL patency in Stanford type A aortic dissection (TAAD) postoperative anticoagulation with an OR of 1.82 (95% CI: 1.22 to 2.71; *Z* = 2.95; *I*²=0%; *P *=* *0.93). Moreover, there was no statistically significant difference between the two groups in aorta-related death, aortic reintervention, and perioperative stroke with an OR of 1.31 (95% CI: 0.56 to 3.04; *Z* = 0.62; *I*² = 0%; *P *= 0.40), 0.98 (95% CI: 0.66 to 1.47; *Z* = 0.09; *I*² = 23%; *P *= 0.26), 1.73 (95% CI: 0.48 to 6.31; *Z* = 0.83; *I*² = 8%; *P *= 0.35), respectively.

**Conclusions:**

Postoperative anticoagulation was associated with higher FL patency in Stanford type A aortic dissection patients. However, there was no significant difference between the anticoagulation and non-anticoagulation groups in terms of aorta-related death, aortic reintervention, and perioperative stroke.

## 1. Introduction

Acute aortic dissection (AAD) is a life-threatening disease that poses a significant challenge for cardiovascular surgeons. Depending on whether the dissection involves the ascending aorta, the Stanford system classifies AAD into Type A (involved) and Type B (not involved). Emergency surgery for type A acute aortic dissection (TAAD) and thoracic endovascular aortic repair (TEVAR) or medical therapy for type B acute aortic dissection (TBAD) were usually indicated, while mortality rates differ significantly (1, 2). The International Registry of Aortic Dissection reveals that the in-hospital mortality rate for TAAD and TBAD are 22% and 13%, respectively (3). Despite advancements in surgical and endovascular procedures and perioperative management, the mortality rate associated with AAD remains a significant concern. Factors such as a larger aortic diameter, extensive false lumen area, young age, Marfan syndrome, and limb malperfusion have been identified as strong predictors for the requirement of late reoperation (4). The status of the FL after AAD repair is of growing concern. A meta-analysis (5) demonstrated that residual patent false lumen is an independent predictor of long-term mortality and aortic events. Kim et al. found that a high distal aortic maximum false lumen area after AAD repair is an independent risk factor for aortic dilation and aorta-related reintervention and is associated with proximal descending thoracic aorta reentry tears after repair of TAAD (6). In addition, one study (7) has suggested that proximal FL thrombosis reduced FL pressure and protected against acute complications and adverse aortic events in TBAD.

Postoperative anticoagulation therapy for AAD is commonly used in patients with atrial fibrillation or mechanical valve implantation. Anticoagulation therapy may theoretically delay false lumen thrombosis and promote false lumen patency, leading to adverse effects on aortic remodeling. However, Vendramin et al. (8) observed that postoperative anticoagulation on TAAD increased FL patency and did not increase the risk of late mortality or reoperation. The early use of anticoagulation prevented partial thrombosis in the residual FL and might provide a more favorable long-term prognosis after the repair of TAAD (9). Moreover, anticoagulation didn't negatively influence FL thrombosis, aortic reintervention, and mortality after TEVAR for TBAD (10). Currently, the effects of postoperative anticoagulation on AAD are not well understood and there are no clear guidelines for postoperative anticoagulation in AAD.

This systematic review with meta-analysis is the first to evaluate the impact of postoperative anticoagulation on TAAD or TBAD patients. Our aim was to conduct a systematic review of the research on postoperative anticoagulation in AD to assess differences in FL status, aorta-related death, aortic reintervention, and perioperative stroke between postoperative anticoagulation and non-anticoagulation individuals, which could guide the postoperative anticoagulation therapy in the clinical management of AAD.

## 2. Methods

### 2.1. Data sources and searches

Comprehensive searches of PubMed, Cochrane Libraries, Embase, and Web of Science were carried out independently by two experienced specialists. Only English-language publications were included in search returns. Related valuable research were manually searched for additional studies. This systematic review and meta-analysis followed the Preferred Reporting Items for Systematic Reviews and Meta-Analyses (PRISMA) guidelines.

### 2.2. Study selection and data extraction

The references were assessed independently by two investigators. The study was eligible if it met the following PICOS criteria: (1) patients: patients among included studies met the diagnosis criteria of TAAD or TBAD; (2) intervention: postoperative anticoagulation; (3) comparison: postoperative non-anticoagulation; (4) outcome: the status of false lumen (FL) (5) study design: clinical controlled trials. Exclusion criteria included the following: (1) studies of chronic aortic dissection; (2) studies including non-surgical treatment of the population; (3) studies in which the average population age was less than 18 years; (4) case reports, editorials, and review articles. Any Discrepancies between the two investigators were resolved by a consensus process.

### 2.3. Quality assessment

Each included study was carefully evaluated by two independent professionals, based on population, study selection, outcome, and comparability. Two authors screened 7 high-quality non-randomized controlled studies according to standard procedures. The Newcastle-Ottawa Scale (NOS) score was employed to evaluate 7 non-randomized controlled studies, with a score ≥ 7 indicating the absence of substantial bias. Funnel plots were used to assess publication bias. The funnel plot was symmetrical which illustrated no significant publication bias.

### 2.4. Study endpoints

The primary outcome of this study was the status of FL, which included patent FL and FL thrombosis. Secondary outcomes included the following endpoints: aorta-related death, aortic reintervention, and perioperative stroke.

### 2.5. Statistical analysis

We analyzed the difference between the postoperative anticoagulation group and non-anticoagulation group in FL status, aorta-related death, aortic reintervention, and perioperative stroke. The population included TAAD and TBAD, developed by subgroup analysis. The odds ratio (OR) and 95% confidence interval (CI) were used to describe dichotomous variables. We applied I² statistic to assess the heterogeneity of the included studies. The fixed effect model or random effect model was chosen according to heterogeneity. The overall effect was determined by *Z* test. Review Manager version 5.3 (The Cochrane Collaboration, Copenhagen, Denmark) was employed for statistical analysis.

## 3. Results

### 3.1. Included studies and characteristics

**Figure 1:**
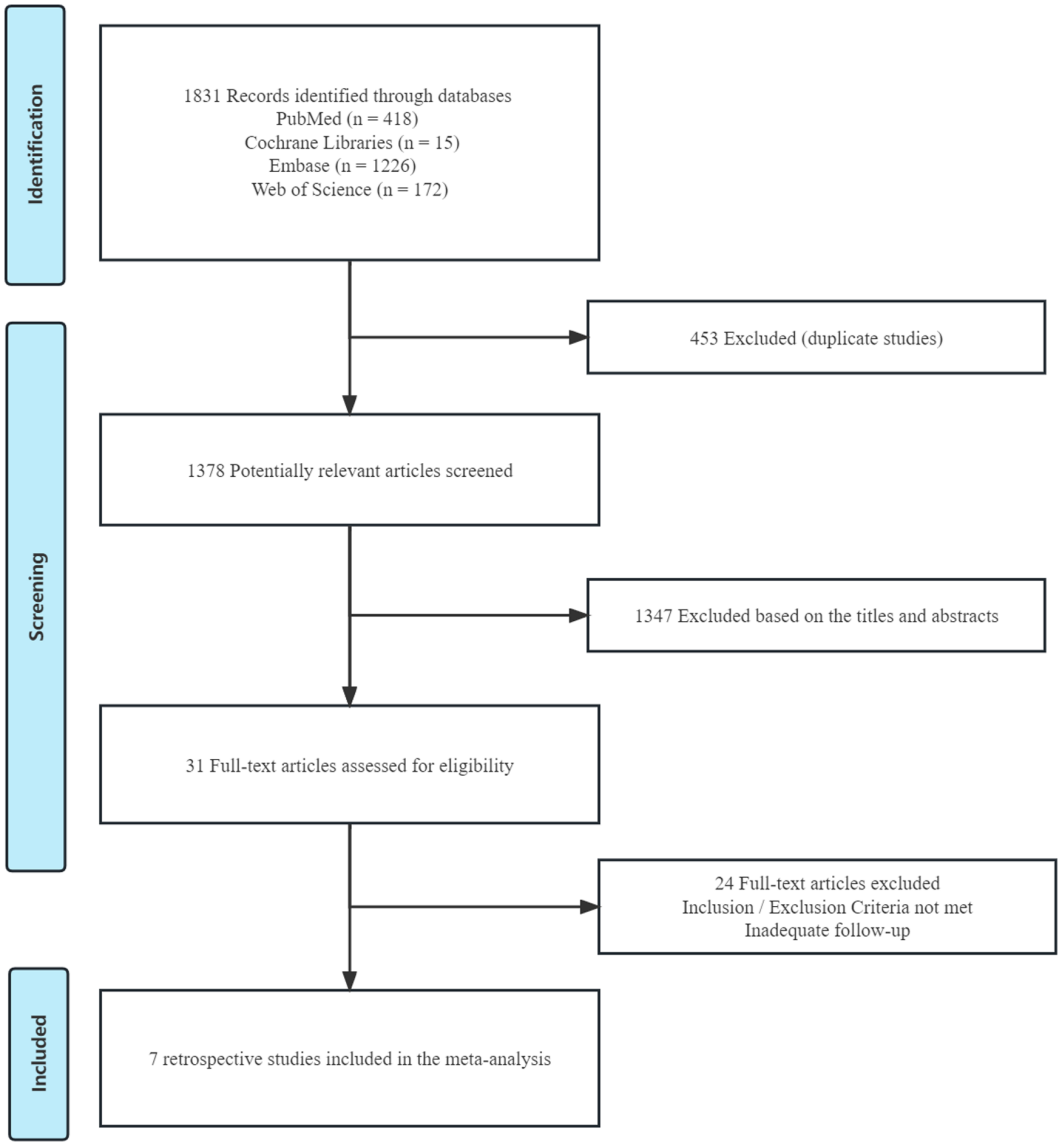
Flowchart showing selection of studies.

**Figure 2:**
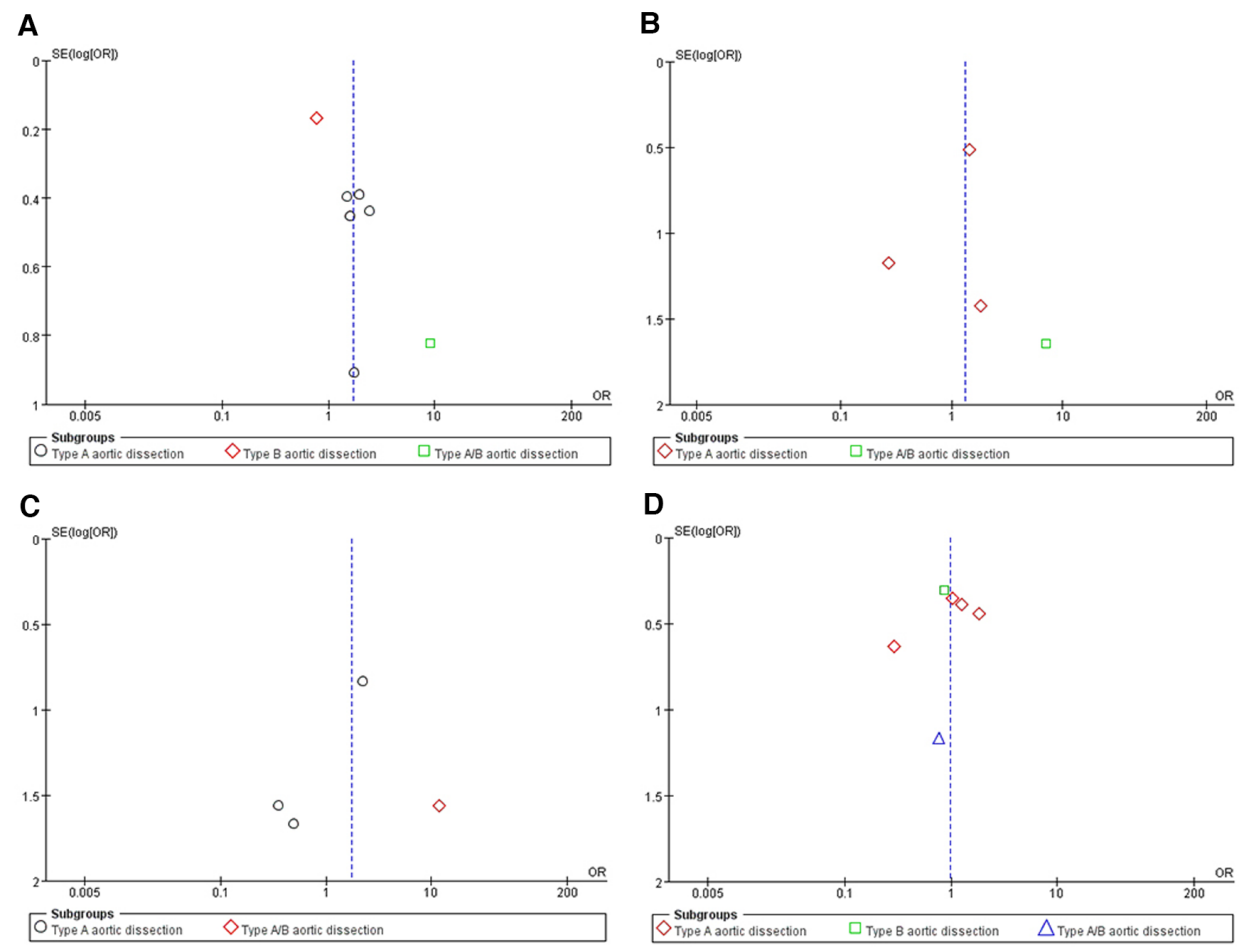
An Egger’s funnel plot indicated low level of heterogeneity for evaluating four outcomes: false lumen patency (**A**), aortic-related death (**B**), perioperative stroke (**C**) and reintervention (**D**)

A total of 1,831 articles was yielded from searches in PubMed, EmBase, and the Cochrane Library, of which 7 retrospective studies (8–14) with 2,122 patients were included for the further quantitative analysis. The Preferred Reporting Items for Systematic Reviews and Meta-Analyses (PRISMA) flow chart provided detailed screening of literature and study selection processes as shown in Figure 1. The risk of publication bias was shown in Figure 2.

Study characteristics were fully summarized in Suppementary Material Table S1. The included retrospective studies cover a publication period from 2010 to 2021. Five studies included patients with TAAD, one study enrolled patients with TBAD and the remaining one study with TAAD and TBAD. The average age of enrolled patients was 42.8 to 65.5 years. Included studies compared postoperative anticoagulation group and non- anticoagulation group in FL. Four articles mentioned aorta-related death and perioperative stroke in both groups. A comparison of the aortic reintervention between two groups was reported in 6 studies.

### 3.2. Primary outcome

**Figure 3:**
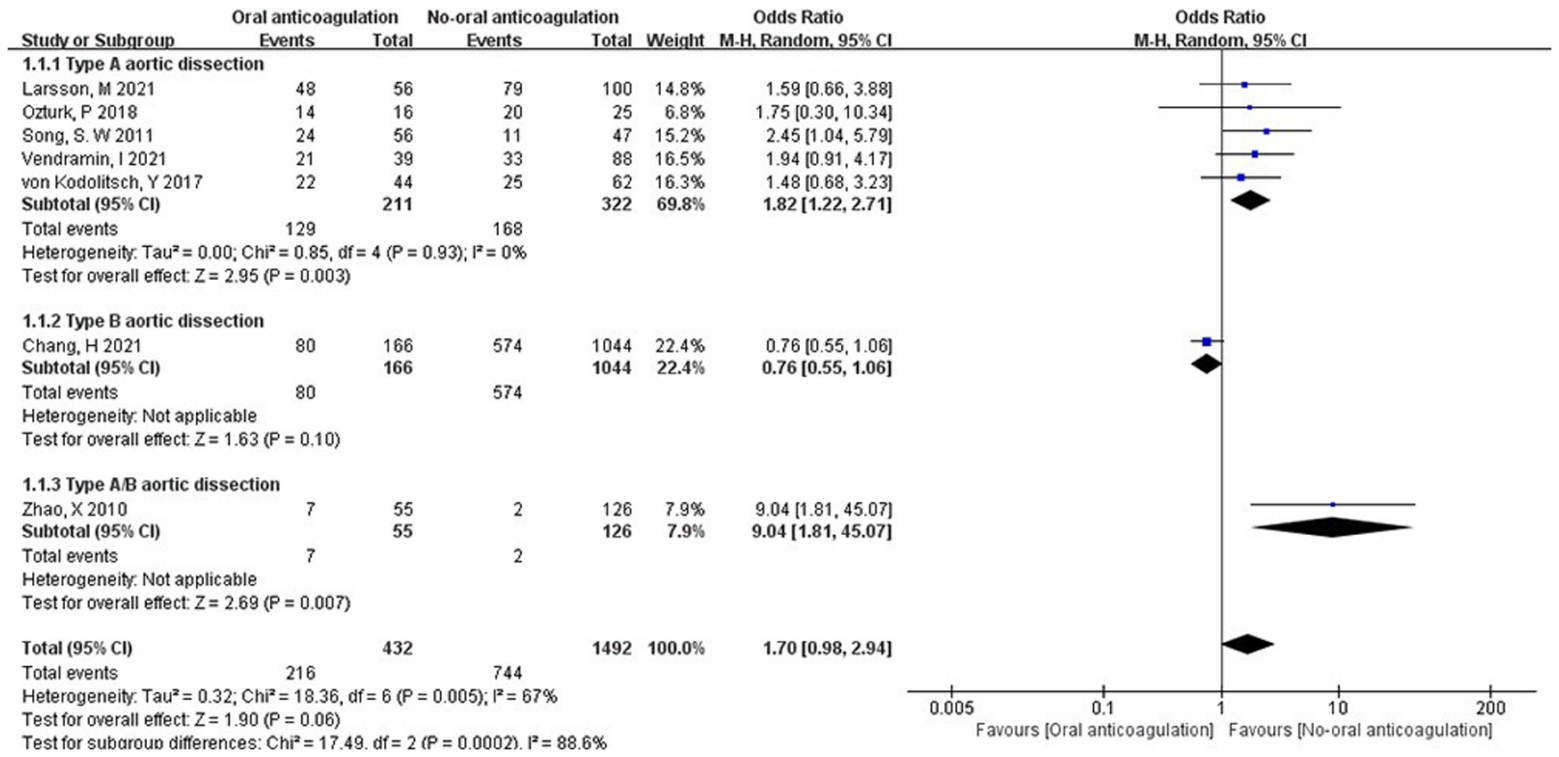
Forest plot showing the HR and 95% CI of false lumen patency for studies comparing the anticoagulation and non- anticoagulation after surgery. HR, hazard ratio; CI, confidence interval.

***The status of FL.*** All the included studies presented the outcome of postoperative anticoagulation vs. non-anticoagulation in the status of FL. The FL patency was 12.7% to 87.5% in postoperative anticoagulation group, and it was 42.9% to 87.5% in TAAD patients. In postoperative non-anticoagulation group, the FL patency ranged 1.6% to 80.0%, and it was 23.4% to 80.0% in TAAD patients. Overall, the pooled OR of postoperative anticoagulation group vs. non-anticoagulation group was 1.70 (95% CI: 0.98 to 2.94; *Z* = 1.90; *I*² = 67%; *P *=* *0.05; Figure 3). Conversely, postoperative anticoagulation was associated with significantly higher FL patency with an OR of 1.82 (95% CI: 1.22 to 2.71; *Z* = 2.95; *I*² = 0%; *P *=* *0.93; Figure 3) in TAAD subgroup. Additionally, the analysis of subgroups revealed an increase in FL patency among patients with postoperative anticoagulation who had undergone treatment for TAAD. Remarkably, Chang, H et al. (10) found that the postoperative anticoagulation of patients with TBAD did not adversely affect the status of FL.

### 3.3. Secondary outcomes

**Figure 4:**
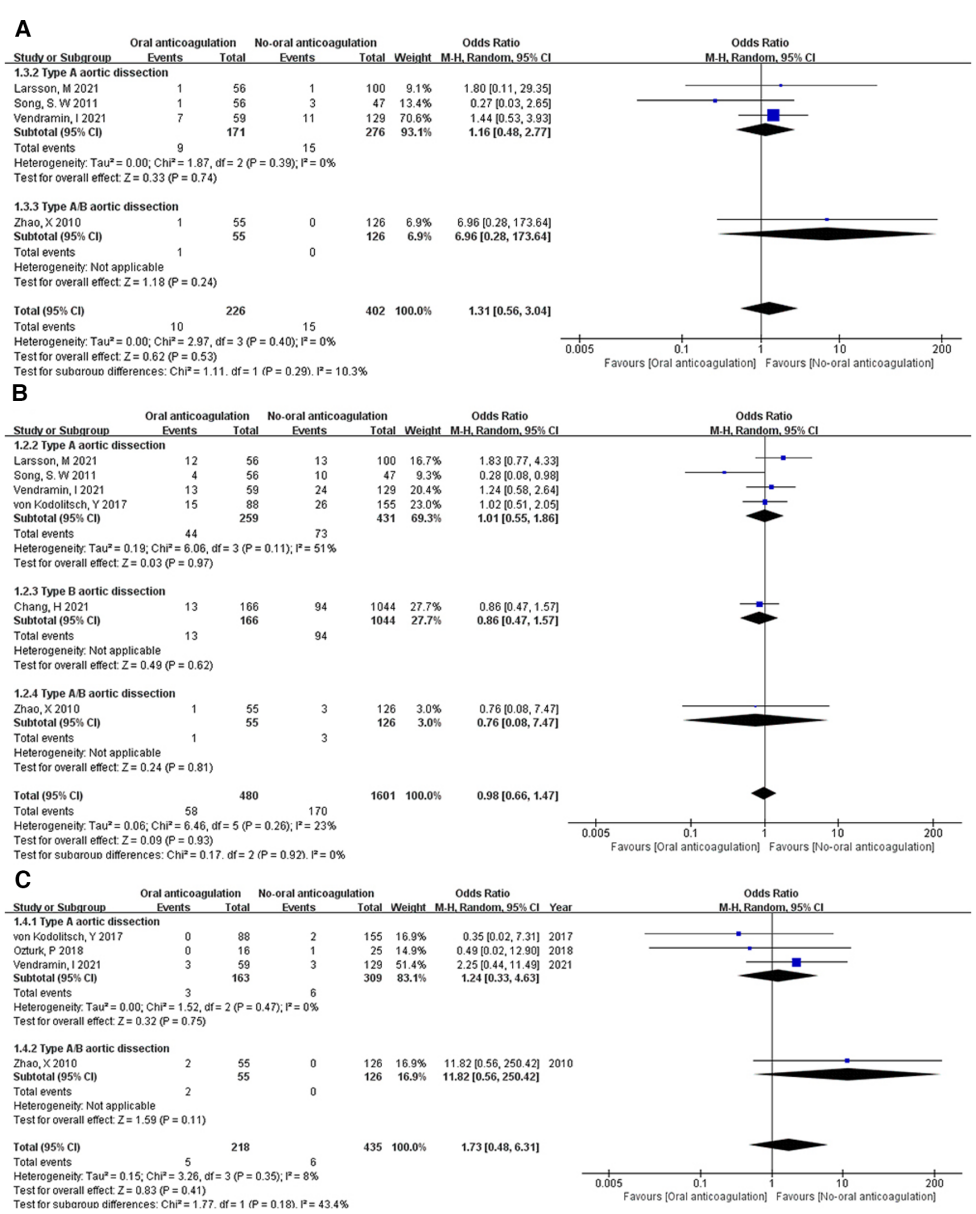
Forest plot showing the HR and 95% CI of aortic-related death (**A**), perioperative stroke (**B**) and reintervention (**C**) for studies comparing the oral anticoagulation and non-oral anticoagulation after surgery. HR, hazard ratio; CI, confidence interval.

***Aorta-related death.*** Three studies (8, 9, 14) reported death of aorta-related complications in TAAD patients, one (11) was reported in TAAD/TBAD patients. In the postoperative anticoagulation group, 10 of 226 (4.42%) patients died of aorta-related complications. In the postoperative non-anticoagulation group, 15 of 402 (3.73%) patients died of aorta-related complications. Interestingly, Song, S. W et al. (9) reported that 1 of 56 (1.79%) TAAD patients died of aorta-related complications in the postoperative anticoagulation group and 3 of 47 (6.38%) TAAD patients died of aorta-related complications in the postoperative non-anticoagulation group. In this study, aorta-related death of postoperative non-anticoagulation group is numerically higher than postoperative anticoagulation group. The OR of aorta-related death between postoperative anticoagulation and postoperative non-anticoagulation group was 1.31 (95% CI: 0.56 to 3.04; *Z* = 0.62; *I*² = 0%; *P *=* *0.40; Figure 4A). No statistically significant difference was detected in the aorta-related death between the postoperative anticoagulation and non-anticoagulation group.

***Aortic reintervention.*** Aortic reintervention was reported in 6 studies (8–12, 14). The forest plot showed no statistically significant difference between the postoperative anticoagulation and non-anticoagulation group in aortic reintervention with an OR of 0.98 (95% CI: 0.66 to 1.47; *Z* = 0.09; *I*² = 23%; *P *=* *0.26; Figure 4B). Furthermore, Song, S. W et al concluded that postoperative anticoagulation in TAAD patients might have a favorable effect on the reintervention.

***Perioperative stroke.*** Four of 7 studies (8, 11–13) reported perioperative stroke in the postoperative anticoagulation and non-anticoagulation group. There was no significant difference in perioperative stroke between the postoperative anticoagulation and non-anticoagulation group with an OR of 1.73 (95% CI: 0.48 to 6.31; *Z* = 0.83; *I*² = 8%; *P *=* *0.35; Figure 4C).

## 4. Discussion

Although previous investigations have sought to examine the effects of anticoagulation therapy in the postoperative setting of AAD, the available evidence has failed to provide a clear consensus on the optimal approach, with no existing meta-analysis to synthesize the results. In light of this, the present study aims to conduct a systematic review with meta-analysis of the patient populations undergoing surgery for AAD and receiving either anticoagulation or non-anticoagulation treatment in order to better inform clinical practice. Our meta-analysis of seven nonrandomized controlled trials, which included a total of 2,122 patients diagnosed with AAD, provides compelling evidence to support the correlation between postoperative anticoagulation and a significantly higher rate of FL patency. However, no statistically significant differences were observed between postoperative anticoagulation and non-anticoagulation patients when considering aorta-related mortality, the need for aortic reintervention, and perioperative stroke in the meta-analysis of the seven studies.

Prognosis is quite heterogeneous between different false lumen status. Postoperative false lumen patency is a serious complication that can occur after surgical repair of aortic dissections, which is recognized as a risk factor for late reintervention and mortality (5, 6, 13). The literature reports the frequency of FL patency after TAAD surgery to be between 42.9% and 87.5%. The pathogenesis of postoperative FL patency is multifactorial, including proximal or distal breach incompletely closed, hypertension, preoperative descending thoracic aortic diameter of ≥35 mm, lower BMI and/or BSA (13, 15, 16). Our study has revealed that oral anticoagulation therapy may lead to an elevation in the incidence of false lumen patency in TAAD patients only, a finding that has been met with significant debate. The potential mechanisms underlying the ability of oral anticoagulation to reduce the occurrence of false lumen thrombosis may be attributed to inhibit thrombin formation and decrease the viscosity of blood (17, 18). However, this difference is not pronounced in patients with TBAD. We recommend the implantation of a frozen elephant trunk stent after intraoperative artificial vascular replacement to maximize coverage of the distal tear, thereby reducing the patency rate of the false lumen and accelerating thrombosis (19–21). In our analysis, the pooled results suggested that postoperative anticoagulation did not have a negative influence on aorta-related mortality, the need for aortic reintervention, and perioperative stroke compared to non-anticoagulation therapy. According to previously published studies, there are several potential explanations for the disagreement. First, strict control of blood pressure and heart rate has become a routine component of postoperative management, as it can help alleviate the pressure in the false lumen and slow its expansion, ultimately reducing the risk of false lumen rupture (4, 22, 23). Moreover, reentrant tearing of the distal abdominal aorta and FL side branches are common causes of FL patency, for which nonsurgical interventions have shown limited efficacy in promoting thrombosis. However, monitoring postoperative coagulation indicators can aid in balancing the risks of bleeding and embolism (14, 21, 24, 25). Consistently, rapid aortic growth predicts late mortality and late aortic events, but warfarin anticoagulation is not associated with aortic growth directly (12).

Due to frequent involvement of the aortic valve in aortic dissection, aortic valve replacement (AVR) surgery is a common intervention for many affected patients. Most patients diagnosed with AD were found to be below the age of 50 and showed a preference for a mechanical valve instead of a biological prosthesis (11). As a result, prolonged anticoagulation therapy post-surgery became a necessity, although clot formation within the false lumen plays a crucial role in the aortic wall remodeling process following repair surgery. While our analysis indicates that postoperative anticoagulation is safe, efforts should be made to minimize the patency rate of the false lumen and promote complete thrombosis of the false lumen. There is clear observational evidence that depressurization and placement of the stent-graft to cover the primary tear promotes false lumen thrombosis and remodeling of dissected aorta (4, 14, 19, 20, 26).

Partial thrombosis is considered the most dangerous of the three thrombotic status. The risk of death in these patients increased by a factor of 2.7 compared to patients with patent false lumen (18, 27). In 2007, Tsai et al. (18) first described the effect of partial thrombosis on the TBAD patients, partial thrombosis of the FL was identified as the strongest independent predictor of postdischarge mortality. The mechanism underlying the higher mortality risk associated with partial thrombosis of the false lumen has been extensively explored in the literature and is generally attributed to hemodynamic and hypoxia/inflammatory theories (7, 22, 26, 27). These theories have been widely accepted in the medical community. In a patent FL, blood flow can perfuse the proximal entry tear and outflow from the distal reentry tear for decompression. Formation of a partial thrombus may occlude these distal tears, impeding outflow and even resulting in a blind sac with a marked increase in the diameter and pressure of the false lumen, posing a significant challenge for preserving the integrity of the aorta. Some scholars even suggested that postoperative FL patency may even be a relatively good predictor of long-term prognosis in patients with TAAD (5). Consistent with all included studies, pooled results showed that partial false lumen thrombosis was less commonly seen in the anticoagulation group. Thus, we believe that postoperative anticoagulation is safe, acceptable and necessary in certain situations such as concomitant atrial fibrillation or mechanical valve replacement.

A partial thrombosis FL has the potential to evolve into patent FL and complete thrombosis and the evaluation of FL status based on single CT images rather than a synthesis of different time points would certainly influence the truth of the study. Thus, a partially thrombosed false lumen requires more frequent imaging surveillance. More aggressive reintervention strategies like septal fenestration or complete endovascular membrane removal should be considered in patients whose false lumen remains partially thrombosed and the area of involvement does not significantly decrease after a period of optimized antihypertensive control (22, 28).

Our study offers novel insights into the management of TAAD, as previous studies did not reach a consensus on the optimal approach to postoperative anticoagulation in TAAD patients. The results may inform clinical decision-making and contribute to the development of treatment guidelines for TAAD patients undergoing surgery. However, it should be noted that the meta-analysis is based on non-randomized controlled trials and therefore, the results may be subject to bias, and further randomized controlled trials are necessary to validate the findings. Anticoagulation-related complications were not counted as study outcomes including gastrointestinal bleeding and cerebrovascular accidents, it is advisable for surgeons to focus on improving their technical skills to optimize aortic valve preservation or to consider utilizing a biological valve that eliminates the need for lifelong anticoagulation (29, 30). Whether systematic extended or total arch replacement for the initial surgical management can decease the incidence of residual patent FL remains huge controversy. The study highlights the need for further research to better understand the mechanisms underlying the relationship between postoperative anticoagulation and partial thrombosis, as well as to determine the most effective strategies for reducing partial thrombosis.

## Data Availability

The original contributions presented in the study are included in the article/**Supplementary Material**, further inquiries can be directed to the corresponding authors.
